# Investigating the effects of red fox management on poultry beyond the controversy, Jura Massif, France

**DOI:** 10.1038/s41598-025-08500-6

**Published:** 2025-07-19

**Authors:** Didier Pépin, Pierre Feuvrier, Thibaut Powolny, Patrick Giraudoux

**Affiliations:** 1 France Nature Environnement 25, Maison de l’Environnement, 25000 Besançon, France; 2 Fédération départementale des chasseurs du Doubs, 25360 Gonsans, France; 3 Chrono-Environnement, Université Marie et Louis Pasteur/CNRS, 25030 Besançon, France

**Keywords:** Adaptive management, Wildlife–human conflicts, Pest control, Research-action, Fox culling, Multi-stakeholders working group

## Abstract

The red fox (*Vulpes vulpes*) is one of the most common generalist mesopredators. Its legal status is often at the centre of controversy between those who wish to protect it and those who wish to control its numbers. In France, foxes are legally hunted and can also be classified as a "species likely to cause damage" (ESOD), the latter status allowing them to be shot and trapped throughout the year. There are a number of legal reasons for this, including damage to poultry. Here, we report the results of a 3.8-year experiment comparing two areas where the fox is protected with areas where it is classified as ESOD. Fox populations were monitored for 4 years before and during the experiment and 231 henhouses were surveyed. 1105 birds were predated in 109 of the 231 henhouses, with an average permanent poultry population of 10,883. 48.3% of damage could be attributed to the category ‘fox’ or ‘likely fox’. Our results show that ESOD status, in the local context, did not lead to a significant reduction in fox numbers or differences in damage rates between areas. However, top and bottom fence protection of outdoor runs significantly reduced fox predation. Better protection for poultry should come from increased security of sheds and outdoor runs, rather than from management of the fox population. The role of dedicated multi-stakeholder working groups for such an evidence-based management at local level is also discussed.

**Supplementary Information:**

The online version contains supplementary material available at 10.1038/s41598-025-08500-6.

## Introduction

The red fox (*Vulpes vulpes*) is one of the most widespread generalist mesopredators. It is considered a pest where it has been introduced in modern times such as Australia, with detrimental impacts on native species. In Western Europe, which is part of its native range in Eurasia, it has co-evolved since hundred thousands of years with the other species of the ecosystems to which it belongs^1^. It preys mostly on small mammals but it can also opportunistically prey on birds, particularly species nesting on the ground^2,3^, on hare (*Lepus europeus*)^4^ and rabbit (*Oryctolagus cuniculus*)^5,6^, be a major cause of neonatal roe deer (*Capreolus capreolus*) fawn mortality^7^ and to lambs^8^ locally. In continental Europe, after being decimated by hunting, trapping, gassing and a rabies epizootic until the 1970s, populations exploded following the massive vaccination campaign of the 1980s and 1990s, but other causes to this population increase are more likely to be found in man-made environmental changes^8^. Fox populations are now larger than before and seem to have generally stabilised, reaching densities close to carrying capacity and even colonising towns, but fluctuate locally due to local epizootics such as sarcoptic mange and distemper.

Foxes are suspected to induce strong predation pressure on their prey species, therefore making them a major target of predator control, particularly in areas where conservation or game hunting programs are applied to a targeted prey species and where attacks on poultry are reported^9^.

The legal status of the fox is often highly controversial^10,11^. Those who want to see the species fully protected argue that it is a predator of small mammal pests, a carrion consumer and, as an important member of a predator community, an indirect regulator of pathogenic organisms such as *Borrelia sp*. responsible for Lyme disease^12,13^. In contrast, people who want to control the species by trapping or hunting argue that fox populations have undesirable impacts on prey species such as game (hare, pheasants) and species of conservation interest (grouses, capercaillie, waterfowl, etc.) and on public health as a propagation agent of the cestode parasite responsible for human alveolar echinococcosis^14,15^ or *Borrelia* sp.^16^. Controversy can also arise from the way in which unwanted effects of a fox population can be limited, with hunting or trapping often argued to be inefficient and/or unethical, and damage to poultry due to inefficient protection rather than fox density^17−19^.

Furthermore, hasty generalisations about fox impacts generally fail to take into account differences between socio-ecosystems, widely varying hunting bags due to hunting traditions^20,21^ and the different prey communities in which these impacts occur, in particular seasonal and interannual variations in prey availability^22^. There are hundreds of websites complaining about red fox attacks on henhouses or dedicated to protecting henhouses, all without clear contextual description of the socio-ecosystem in which they are observed. Moreover, few scientific articles attempt to quantify these attacks in their context (but see Moberly et al.^19^).

In France, the red fox is a game species and can also be classified as a “species likely to cause damage” (“espèce susceptible d’occasionner des dégâts”, ESOD, articles R427-1 to R427-28 of the Environmental Code) ‘in the interests of public health and safety, or to ensure the protection of flora and fauna, or to prevent significant damage to agricultural, forestry and aquaculture activities, or to prevent significant damage to other forms of property’. Damage to poultry is one of the main arguments put forward to justify this classification. This legal status, which allows hunting and trapping all year round under certain conditions, even outside the hunting season, is reviewed every three years at national level on the basis of proposals from the prefects (State-dependent decision authority in France). During these periods, disputes reach a climax and some go as far as the administrative courts. In the Doubs, a consortium of farmers, conservationists, hunters and health professionals, supported by researchers and the administrations, decided to go beyond the controversy and carry out an experimental comparison, called Careli, between areas where the fox is protected and areas where it is classified as an ESOD, in order to obtain objective information on the impacts of this difference, taking into account socio-ecosystems characteristics (https://zaaj.univ-fcomte.fr/spip.php?article115).

This article reports on the survey carried out to investigate fox damage to poultry in these experimental areas and aims to answer the question of whether classifying the fox as an ESOD has a measurable effect on damage to poultry. It was hoped that the results would provide a better understanding on the causes of predation, the effects of the fox legal status on poultry damage, and objectively inform management decisions.

## Material and methods

### Area and sample selection

**Fig. 1:**
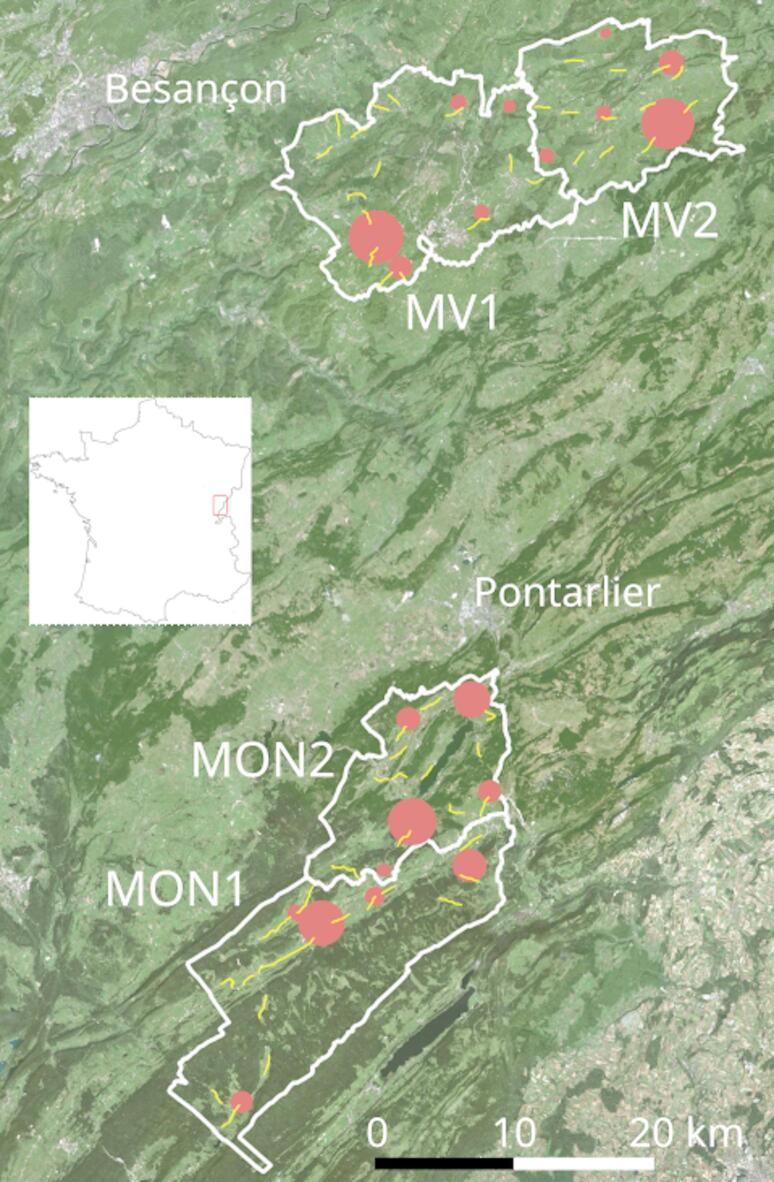
Location of the study areas. MON and MV stand for Mont d’Or—Noirmont and Monts-de-Villers respectively; 1, for fox as ESOD, 2 for fox protected; red circles, villages included in the sample (the size of the circle is proportional to the square root of the population); yellow lines, tracks of the fox night road side counts. Aerial photographs of 2023 from the *Institut Géographique National*. The box in the map indicates the location of the area in France.

The henhouse survey was carried out from April 6, 2021 to January 6, 2025 in two study sites at two different altitudes, corresponding to hunting management units of the *Fédération départementale des chasseurs du Doubs* (FDC25). This period will be referred to hereafter as the Careli period (2021–2024). Fox populations were surveyed from 2017 to 2024. These study sites were selected because, at a given altitude, they presented homogeneous landscapes in terms of both composition (Table 1) and structure (Fig. 1), and were representative of the department’s main socio-ecosystems. Each study site was divided into two areas, one in which fox hunting and trapping were prohibited by prefectorial decree, and the other in which the fox could be hunted and was classified as an ESOD (Fig. 1).

**Table 1:** Land cover and elevation of the study areas.

Habitat (%)	MON1	MON2	MV1	MV2
Arable land	1	3	4	8
Mixed farmland	14	13	27	24
Grassland	19	24	23	33
Broad-leaved forest	0	3	28	20
Mixed forest	17	5	10	8
Coniferous forest	47	39	5	5
Wetland and water bodies	1	8	0	1
Artificial surfaces	2	4	5	2
Total (ha)	22,953	12,676	20,458	13,528
Elevation (m)	MON1	MON2	MV1	MV2
Minimum	884	839	418	475
Maximum	1450	1155	831	949
Mean	1136	958	598	687

MON, Mont d’Or—Noirmont ; MV, Monts-de-Villers ; 1 fox could be hunted and is classified as an ESOD ; 2, fox is protected. Data Corine Land Cover 2018^23^ and DEM of metropolitan France, 100 m resolution^24^.

The main characteristics of those areas are presented in Table 1.

20 villages were included in the survey, distributed as evenly as possible across the study sites, trying to match population size of villages among areas as closely as possible (Table 2). Each village was surveyed by *France Nature Environnement Doubs* (FNE25) first by asking the mayor and then residents about the location of henhouses and poultry farms until no new henhouses could be found. Contact was then made with each owner and the henhouse was described in detail (Supplementary material 1). Every six months thereafter, the owner was contacted to find out the number of birds present and whether or not he had suffered any damage, and if so, a detailed investigation of the damage was carried out using a questionnaire (Supplementary material 2). On this occasion, the owner was also asked if any structural changes had been made to the henhouse. If so, a new description was made. In order to assess the level of protection of each henhouse for each of its descriptions, it was scored on the basis of 10 scored variables (the higher the score, the higher the protection). For the building: ground type of the building, type of closing system, number of openings, for the outdoor run (if any): outdoor run, ground type of the outdoor run, type of fence, minimum height of the fence, protection of the top of the fence, protection of the bottom of the fence and surveillance system (see Supplementary material 3 for details). The sum of the scores was called ‘protection score’ and used to rank the level of protection of the henhouse. The questionnaire and each step of the survey were conducted in accordance with the European GDPR Regulation (2016/679 of the European Parliament and of the Council of 27 April 2016). All experimental protocols were approved by the University Marie and Louis Pasteur and the Prefecture of the Doubs department. Informed consent was obtained from the owners of the henhouses included in the survey.

**Table 2:** Distribution of villages in the study areas.

	MON1	MON2	MV1	MV2
n	5	5	5	5
Minimum	139	105	88	62
Maximum	1133	1231	1586	1446
Median	269	304	140	124
Henhouses	67	70	43	51
Observation duration (days)	49,650	59,635	36,753	43,697

n, number of villages; minimum, maximum and median of population size per village; henhouses, number of henhouses; observation duration, the total number of observation days.

### Fox relative densities, hunting bags and trapping

Fox populations were surveyed by FDC25 from March to April (pre-breeding period) depending on altitude using night roadside counts. Each sampling event consisted of driving a car with 4 people (the driver, a data recorder and two observers) along 54 fixed tracks (range 0.5–4.7 km, mean 1.4 km) totalling 76 km, at less than 20 km/h (Fig. 1). The tracks were chosen because they could be observed equally well from every area, i.e. there were few or no obstacles to distant observation. Observations were performed using 100-W spotlights at night and binoculars for species identification. Sampling was carried out on 3 successive nights after sunset called a ‘session’. A Kilometric Abundance Index (KAI) was calculated for each session as the maximum number of animals recorded km^−1^ (thus providing a lower limit for the number of animals present).

The number of foxes collected was reported each year in June to the FDC25, including the hunting season (September to January) by each local hunting association, and the number of foxes trapped was reported to the *Direction départementale des territoires du Doubs*.

### Statistics

Statistics and graphical displays were performed in R (version 4.4.2) with the packages gamlss^25^, lme4^26^, MASS^27^ and nibble^28^, using QGIS 3.34.14 complementarily. Differences were considered statistically significant for p(Ho) ≤ 0.05.

Fox KAI were compared using generalized linear models with a Poisson or negative binomial error distribution of the general form: log(n) = ln(x_1_) + a_0_ + a_1_x_2_ + ε, with n, the number of foxes observed, x_1_, the length of the track, x_2_, the name of the study area, a_i_, the model coefficients, and ε, the residuals. The logarithm of the length of the track was offset.

Damage intensity was calculated as the number of observed damages per observation period in days. To compare damage intensity between areas, we used generalized linear models with a Poisson or negative binomial error distribution with a random error term for villages of the general form: ln(n) = ln(x_1_) + a_0_ + a_1_x_2_ + a_2_x_3_ + ε, with n, the number of damages, x_1_, the observation duration, x_2_, the study area, x_3_, the village random term, a_i_, the model coefficients, and ε, the residuals. The logarithm of the observation duration was offset. The random term for villages takes into account the fact that there were several henhouses in the same village.

To assess whether the number of damages could be due to the condition of the henhouses, we used generalized linear models with a Poisson or negative binomial error distribution of the general form: ln(n) = ln(x_1_) + a_0_ + a_1_x_2_… + a_p_x_p_ + ε, with n, the number of damages, x_1_, the observation duration, x_2_ to x_p_, the variables describing the henhouse condition (see Supplementary material 3), a_i_, the model coefficients, and ε, the residuals. The logarithm of the observation duration was offset.

All models were compared using AIC following Burnham and Anderson^29^. The parameters and credible intervals of selected models were calculated using a Bayesian approach^30^.

Data are available at 10.5281/zenodo.14956599.

## Results

231 henhouses were surveyed with a total of 189,735 observation days (Table 2). In average, 73% of these were family holdings with less than 10 birds and 1.2% were poultry farms with more than 99 birds (Table 3). Moreover, these farms produced 82% of the total poultry surveyed.

**Table 3:** Number of henhouses by size class of poultry stock.

	0–9	10–19	20–49	50–99	100–4000
MON1	55	10	1	0	1
MON2	61	8	1	0	0
MV1	27	11	5	0	0
MV2	30	15	4	0	2

Table 4 shows the distribution of the average number of birds per species.

**Table 4:** Average bird numbers by species.

Species	Average number	Percentage
Chicken	10,044	92.3
Guinea fowl	287	2.6
Turkey	260	2.4
Pigeon	182	1.7
Duck	65	0.6
Goose	19	0.2
Quail	14	0.1
Others	12	0.1
Total	10,883	100.0

### Fox population

We did not find statistical differences in KAI between MON1 and MON2 with an average KAI of 2.0 foxes.km^−1^ (95% credibility interval: 1.8–2.3) in the two areas during the Careli period (2021–2024). There was also no statistical difference between MON1 and MON2 in the 2017–2020 period before the experiment.

During the Careli period, fox KAI was more than 1.5 times larger (95% confidence interval 1.2–1.9) in MV2 than in MV1 with an average KAI of 1.5 foxes.km^−1^ (95% credibility interval 1.3–1.8) in MV1 (ESOD area) and 2.4 foxes.km^−1^ (95% credibility interval 2.1–2.7) in MV2 (protected area). A similar difference was observed during the period 2017–2020 preceding the experiment, with MV2 KAI 1.5 times larger than MV1 (95% credibility interval 1.1–2.1). Hence, the same IKA difference between MON1 and MON2 had already been observed during the four years prior to the experiment, despite the two areas having the same status at that time (no fox protection). During the experiment, this difference did not change.

Table 5 shows the number of foxes killed in the study areas, indicating the hunting and trapping pressure on foxes in areas where they are an ESOD four years before (2016–2020) and during (2020–2024) the experiment.

**Table 5:** Number of foxes shot or trapped in each area.

	MON1		MON2		MV1		MV2	
Period	Shot	Trapped	Shot	Trapped	Shot	Trapped	Shot	Trapped
2016/2017	32	3	28	1	43	1	45	10
2017/2018	22	3	23	0	45	9	12	11
2018/2019	54	0	29	0	102	0	39	7
2019/2020	52	0	36	0	57	3	11	0
2020/2021	19	0	0	0	58	3	0	0
2021/2022	36	0	0	0	64	5	0	0
2022/2023	41	0	0	0	73	0	0	0
2023/2024	60	0	0	0	98	3	0	0
Total	316	6	116	1	540	24	107	28

### Damages

Damage questionnaires where carefully examined by two wildlife specialists (DP and Dominique Michelat) and damage was categorized in 18 predator categories (Table 6 and Supplementary Material 4 for criterions).

**Table 6:** Number of damages in each category.

Species	MON1	MON2	MV1	MV2	Total	%
Likely fox	14	9	6	9	38	16.6
Fox or dog	14	7	8	7	36	15.7
Fox	14	8	3	6	31	13.5
Unidentified	11	10	3	5	29	12.7
Likely goshawk	10	4	6	3	23	10.0
Likely stone marten or other mustelids	6	5	6	6	23	10.0
Dog	7	6	2	0	15	6.6
Unidentified carnivore	1	6	1	3	11	4.8
Goshawk	1	0	4	1	6	2.6
Likely dog	1	1	1	0	3	1.3
Stone marten	0	0	1	2	3	1.3
Unidentified bird of prey	1	1	1	0	3	1.3
Common buzzard	2	0	0	0	2	0.9
Unidentified corvid	1	0	0	1	2	0.9
Likely goshawk or stone marten	1	0	0	0	1	0.4
Human	0	1	0	0	1	0.4
Pine marten	0	0	0	1	1	0.4
Fox or stone marten	1	0	0	0	1	0.4
Total	85	58	42	44	229	100

During the study period (3.8 years), 1105 birds have been predated in 109 (47%) of the 231 henhouses surveyed (Table 7), representing 2.7% (1105/10,883/3.8) each year of the average bird number present in henhouses. The large number of kills in MV2 was explained by two major damage in professional chicken farms and two in two private pigeon farms. Furthermore, 483 of the 1105 birds (43.7%) were taken from 81 henhouses among the 173 henhouses with less than 10 birds (46.8%).

**Table 7:** Number of birds caught by predators.

Area	Killed	Eaten on site	Taken away	Wounded
MON1	86	35	138	8
MON2	41	8	79	6
MV1	93	26	87	6
MV2	408	8	75	1
Total	628	77	379	21

Table 8 shows the distribution of birds killed by predators. 48.3% could be attributed to the category ‘fox’ or ‘likely fox’.

**Table 8:** Number of birds caught (wounded included) by predator category.

Species	MO1	MO2	MV1	MV2	Total	%
Fox	60	47	25	244	376	34.0
Likely stone marten or other mustelids	35	6	44	92	177	16.0
Likely fox	44	25	36	53	158	14.3
Fox or dog	41	10	62	29	142	12.9
Unidentified	17	13	6	11	47	4.3
Likely goshawk	29	4	8	3	44	4.0
Dog	16	16	7	0	39	3.5
Pine marten	0	0	0	33	33	3.0
Unidentified carnivore	3	8	12	4	27	2.4
Stone marten	0	0	5	20	25	2.3
Goshawk	4	0	5	2	11	1.0
Common buzzard	10	0	0	0	10	0.9
Fox or stone marten	4	0	0	0	4	0.4
Likely dog	1	1	1	0	3	0.3
Unidentified bird of prey	1	1	1	0	3	0.3
Human	0	3	0	0	3	0.3
Unidentified corvid	1	0	0	1	2	0.2
Likely goshawk or stone marten	1	0	0	0	1	0.1
Total	267	134	212	492	1105	100

The seasonality of damage is shown in Table 9. The null hypothesis of a random distribution between months could be rejected for the two categories (Chi-squared test, p < 0.007). Damage attributed to fox was higher from March to August, and was more irregularly distributed in the other categories, but also with higher values from March to August and an isolated peak in November.

**Table 9:** Damage seasonality.

Month	1	2	3	4	5	6	7	8	9	10	11	12
Fox or likely fox	1	3	8	9	9	15	3	8	4	3	0	6
Others	10	7	24	16	12	17	15	18	10	6	18	7

### Damage all causes included

Damage was significantly 1.8 times higher (95% credibility interval = 0.9–3.3, p(risk ratio ≤ 1) = 0.04) in MON1 (ESOD) than in MON2 (protected). We did not detect a difference between MV1 and MV2 (Supplementary material 5).

### Damage attributed to foxes only

For the categories ‘fox’ and ‘likely fox’ collapsed, we did not find statistically significant difference between the two areas of the two study sites (Supplementary material 5).

### Ultimate causes and henhouses

#### Intrusion types

Table 10 shows the types of intrusions identified for the category ‘fox’ or ‘likely fox’. 82% of the intrusions are due to human negligence (41%), insufficient shelter (36%) or poultry out of the pen (5%), and 18% are due to fox break-in.

**Table 10:** Types of intrusion for the category fox or likely fox.

Access	Method	Cat	n	%
To the fenced area around the henhouse	Digging a hole	A	13	20
	Damaging the fence	A	3	4.6
	Going over the fence	C	24	36.9
	Passing under the fence	C	3	4.6
	Going through the fence	C	10	15.4
	Through an access left open	B	6	9.2
	Entering without breaking	C	1	1.5
	The chickens went out of the pen	D	5	7.7
	Undetermined*		4	
	Total identified		65	100
To the henhouse	Digging a hole	A	2	4.8
	Damaging part of the building	A	2	4.8
	Through an access left open	B	22	52.4
	Following a malfunction of the automatic locking system	B	4	9.5
	Automatic (or manual) closing before the birds enter	B	12	28.6
	Undetermined**		27	
	Total identified		42	100

Cat, category: A, predation by breaking, B, predation due to human negligence, C, predation due to inadequate shelter, D, poultry outside the pen. * 3 unspecified predations and 1 predation in a farm without an outdoor enclosure, ** 19 predations during the day (= open building), 4 predations with chicken outside the pen and 4 predations at night but no details.

#### Henhouse condition

We found no statistical differences in henhouse protection scores between zones and study sites (Supplementary material 5). Based on the scoring criteria, theoretical protection scores range from a minimum of 4 to a maximum of 63. The average protection score was 27.2 (95% bootstrapped confidence interval 26.4–28.1, range 15.7–46.3).

In order to assess the contribution of each of the variables describing the conditions of henhouse against fox predation, we compared henhouses where predation was ‘fox’ or ‘likely fox’ to henhouses where no predation was reported (n_1_ = 52, n_2_ = 126, respectively). Of the 10 variables used, we found that only top and bottom fence protection significantly reduced the fox predation events by 10% (95% credibility interval 1.7–19.8) and 11% (95% credibility interval 1.7–21.1) respectively (Supplementary material 5).

## Discussion

To the best of our knowledge, this study is the first to address the issue of the effectiveness of legal fox control in preventing damage to poultry, including both non-commercial and commercial flocks, based on a comprehensive inclusion of henhouses and poultry farms in each of the selected villages.

Moberly et al.^19^ analysed self-selected responses to a questionnaire sent to industrial free-range poultry farms (return rate 34%). Relative densities of foxes were estimated as the total number of scats recorded on transects for nine regions. In this study, the extent of predation was reported to be less than 2% for all producers with marked differences between them. The extent of predation was not associated either with the large-scale density of the fox population or with the variations in the farms’ habitat, and there was a positive association between losses due to other causes and chicken predation. This study was compared to earlier enquiries targeting non-commercial producers indicating that commercial producers tend to lose fewer birds to fox predation and are less likely to experience fox predation than small-scale poultry keepers^21,31^. Moberly et al.^19^ concluded that henhouse management would be the most cost-effective means of reducing fox predation rather than greater fox control.

Of the farms in our study areas, only three were commercial chicken farms with relatively large numbers of birds. Almost three quarters were small family henhouses with fewer than 10 birds, and 98.8 per cent had fewer than 50.

In our study, fox relative densities were estimated using night road side counts. This method does not allow absolute densities to be estimated directly and without biases, but a study using distance sampling in the same region suggests that a conversion coefficient of 2.5 can be used for a rough estimate of these densities based on KAI^22^. This gives an order of magnitude of 3.8 to 6 foxes.km^-2^ in early spring in the open areas of the landscape that could be surveyed using this method. Compared to other areas in Europe^9^, fox population densities could therefore be considered high, with several hundred foxes per site in these habitats (approximately 380 in MON1, 250 in MON2, 410 in MV1, 520 in MV2). The proportion of foxes collected or trapped in areas where foxes were classified as ESOD, as an order of magnitude, was probably less than 10% of this population in MON1 and could reach 16% in MV1. However, this culling pressure was not enough to make a significant difference of KAI between MON1 and MON2. Moreover, foxes were classified as ESOD in the two areas prior the experiment, and the difference in culling pressure between MV1 and MV2 (protected) was not sufficient to create a greater difference between MV1 and MV2 between the four years prior to the experiment and the experiment period, and to limit the population growth observed in the two areas.

A larger number of predator species could be considered responsible for predation on poultry in all areas, and foxes accounted for at least 30% of the predation events and at least 48% of the kills, probably more given identification uncertainties (see Table 6). The overall proportion of poultry taken can hardly be estimated without knowing the turnover of the flocks.

Our study shows either no association between the ESOD status for fox and damage, regardless of the ecosystems (middle mountains or lower), or counter-intuitively a significant excess of damage in MON1, one of the areas where foxes were ESOD (we could not explain this).

Many studies suggest that fox populations can control prey populations like Iberian rabbit^5^ or European hare^22^ and some experimental studies have shown that fox control can be associated with larger prey populations e.g. hare in Poland^4^ or woylies (*Bettongia penicillata*) and cats (*Felis catus*) in Australia^32^. However, the effect of fox control in a predator community is not easily predicted, as shown in the latter study, where the reduction in fox numbers was offset by an increase in cat numbers and their predation on woylies, thus reducing the effectiveness of fox control programmes on prey species. Furthermore, it appears that, at least in areas where the fox population is relatively large, recreational hunting and even culling are generally ineffective in achieving a significant reduction in population density, as has been demonstrated locally in an experiment to control the transmission of the *E. multilocularis* parasite in eastern France^17^. On the other hand, a massive reduction in the fox population through non-intentional poisoning can have an effect in limiting this parasite transmission^33^.

Our results suggest that ESOD status in the study areas does not result in sufficient culling pressure to have an effect on predation on poultry, given the high level of fox populations and the fact that foxes are generally not specifically hunted or trapped, but are opportunistically shot during other hunts (roe deer, wild boar (*Sus scrofa*), hare, etc.) in our region. The low level at which a fox population should be maintained to prevent damage to poultry is likely to be very low, as shown in the UK, where fox densities as low as 0.5–1.2 foxes.km^-2^ in the pre-breeding period did not limit damage^19,20^, to compare to the 3.8–6 foxes.km^-2^ estimated in our study. Moreover, our study also indicates that better protection of henhouses, with particular attention to the top and bottom of the fence, can reduce fox damages. It is therefore likely that increased protection of henhouses and outdoor runs would be more cost effective and socially acceptable than increased culling of a species in its native range, which is moreover unlikely to be achieved where populations are close to the carrying capacity of their habitats over large areas on a large scale without pharaonic means.

One of the limitations of our study is that the results apply to two typical socio-ecosystems (hunting/trapping and poultry farming traditions, agricultural landscapes, etc.) of the Doubs department, but cannot be generalised without caution to any other type of socio-ecosystem. Here, large populations of common voles (*Microtus arvalis*) and montane water voles (*Arvicola terrestris*), numbering in the hundreds per hectare, can cause problems in grasslands. Farmers consider them to be pests^34–36^. Variations in vole population density shapes the local predator community as shown in earlier studies^22^. During the experiment, the population of montane water voles increased at all study sites. This represents a large and easily accessible source of prey for foxes. Montane water voles and common voles are known to form the bulk of their diet^37^.

Our study confirms the inappropriateness of the ESOD status, as applied locally, for the protection of poultry in such socio-ecosystems. Nevertheless, this status may be granted for other reasons. At national and local level, fox culling has also been judged to be generally inappropriate to protect public health and safety^10,17^. However, in the Careli programme, further evaluations are underway to assess its effectiveness in achieving other objectives invoked in the official regulations to justify an ESOD status (https://zaaj.univ-fcomte.fr/spip.php?article115).

As for other species and the possible damage they cause, it becomes crucial to have well-regulated yet effective control options that have minimal environmental impact^18,38^. These options should include a range of adaptive management strategies^39^ in a ‘toolbox’, primarily aimed at protecting henhouses from predators, and not pointlessly aimed at reducing predator populations where control has been shown to be ineffective, too costly, ethically questionable or technically impossible. It will then be necessary to verify, on the basis of scientific evidence, whether these decisions achieve their objective or whether, as in the present case, they are ineffective in reducing damage. It is therefore essential to have the means to objectively measure the impacts of any option taken in this context, with explicit objectives, and iteratively adjust management options accordingly^39,40^. The existence of dedicated working groups (or multi-stakeholder platforms)^41^, such as Careli, to manage locally, on the basis of scientific evidence, the impacts of species considered as ‘likely to cause damage’ is a crucial point in arriving at acceptable trade-offs between stakeholders weighing up costs and benefits.

## Electronic supplementary material

Below is the link to the electronic supplementary material.

Supplementary Material 1

## Data Availability

Data have been deposited at 10.5281/zenodo.14956599.
