# Supplementary material for: Investigating the effects of red fox management on poultry beyond the controversy, Jura Massif, France
Source: Sci Rep. 2025 Jul 19;15:26238. doi: 10.1038/s41598-025-08500-6 (PMC12276238; doi:10.1038/s41598-025-08500-6)
Supplement: Supplementary file 1 — Supplementary Material 1 [file 41598_2025_8500_MOESM1_ESM.zip › Supplementary_material_1_Fiche descriptive_poulailler.pdf]

Supplementary material 1

Code

Fiche descriptive des élevages avicoles

Vous êtes propriétaire d'un élevage de volailles situé dans une commune du territoire d'étude du programme de recherche CARELI.
Nous vous remercions d'avoir accepté de participer à cette étude en remplissant avec nous (ou sans nous) le questionnaire suivant.
Toutes les parties grisées de ce formulaire nous sont réservées et ne sont donc pas à renseigner.

Les informations portées sur ce formulaire font l'objet d'un traitement informatisé destiné à FNE 25 et seront traitées de façon anonymisée par l'équipe du programme CARELI. Depuis la loi "informatique et libertés" du 6 janvier 1978 modifiée, vous bénéficiez d'un droit d'accès et de rectification aux informations qui vous concernent. Si vous souhaitez exercer ce droit et obtenir communication des informations vous concernant, veuillez vous adresser à mission@fne25.fr - Représentant FNE 25 du programme CARELI.

Nom de la personne ayant renseigné cette fiche :

Date de la description : ....

Caractéristiques de l'élevage

Adresse complète :

Code postal : Commune :

Coordonnées GPS

Longitude :

Latitude :

- Type d'élevage : ☐ Professionnel ☐ Particulier
- Nombre d'animaux à ce jour :

Poule	Dinde	Canard	Pintade	Caille	Oie	Pigeon	Perdrix	....	TOTAL

Caractéristiques du bâtiment d'élevage (où les volailles dorment et pondent)

- Surface (approximative) : m²
- Matériau de construction dominant : ☐ Pierre ☐ Parpaing ☐ Métal ☐ Bois ☐ Plastique ☐ ....
- Année de construction : ....

  ou cocher l'intervalle correspondant : ☐ Moins de 5 ans ☐ Entre 5 et 10 ans ☐ Plus de 10 ans
- Nature du sol : ☐ Terre battue ☐ Pierre ☐ Dalle béton ☐ Bois ☐ ....
- Nombre d'ouvertures : ☐ Porte ☐ Fenêtre ☐ Ouverture réservée aux volailles ☐ ....
- Protection des ouvertures réservées aux volailles contre l'intrusion des prédateurs :
  - ☐ Aucun système de fermeture
  - ☐ Système de fermeture manuel non sécurisé
  - ☐ Système de fermeture manuel sécurisé (crochet, cadenas...)
  - ☐ Système de fermeture automatique (programmateur)

Caractéristiques du parcours extérieur (où les volailles peuvent sortir)

- Les limites du parcours extérieur :
  - ☐ Le parcours est inexistant, les volailles restent toujours enfermées
  - ☐ Le parcours est clos, mais les volailles peuvent sortir ou les volailles sont en liberté
  - ☐ Le parcours est clos et les volailles ne peuvent pas en sortir
- Surface (approximative) : m²
- Nature du sol : ☐ Herbe ☐ Terre battue ☐ Graviers ☐ Bois ☐ Béton ☐ ....
- Type de clôture : ☐ Maçonnerie ☐ Grillage ☐ Bois ☐ Filet non électrifié ☐ Filet électrifié ☐ ....
- Hauteur de la clôture : maximale m minimale m

- **Protection de la clôture :**

	En partie haute	En partie basse
Aucune protection supplémentaire	☐	☐
Fil barbelé	☐	☐
Fil électrifié	☐	☐
Structure recourbée vers l'extérieur de l'enclos	☐	
Grillage enterré : profondeur …………… cm		☐
Grillage recourbé et/ou enterré côté extérieur		☐
Structure béton enterrée : profondeur …………… cm		☐
Parcours extérieur entièrement couvert (filet, grillage, ...)	☐	
Sol entièrement protégé (grillage, dalle béton, ...)		☐
Autre	☐	☐

- Accès de l'éleveur au parcours extérieur par un portail sans passer par le bâtiment d'élevage : ☐ OUI ☐ NON

Si oui, caractéristiques du portail :

- **Nature :** ☐ Bois ☐ Grillage ☐ Métal ☐ ....
- **Hauteur :** [m]
- **Protection du portail :**

	En partie haute	En partie basse
Aucune protection supplémentaire	☐	☐
Fil barbelé	☐	☐
Fil électrifié	☐	☐
Structure recourbée vers l'extérieur de l'enclos	☐	
Grillage enterré : profondeur …………… cm		☐
Grillage recourbé et/ou enterré côté extérieur		☐
Structure béton enterrée : profondeur …………… cm		☐
Parcours extérieur entièrement couvert (filet, grillage, ...)	☐	
Sol entièrement protégé (grillage, dalle béton, ...)		☐
Autre	☐	☐

Environnement proche de l'élevage (quelques dizaines de mètres)

- ☐ Friche ☐ Forêt ☐ Prairie ☐ Arbres fruitiers ☐ Potager
  ☐ Poste de compostage ouvert ☐ Poste de compostage fermé ☐ Tas de fumier ☐ Poubelles
  ☐ Mangeoire à oiseaux ☐ Point d'eau (ruisseau, mare, etc.) ☐ Autre : ....

Dispositif de surveillance

- ☐ Surveillance humaine régulière ☐ Présence d'au moins un chien
  ☐ Répulsif à ultrasons/lumineux/à jet d'eau ☐ Répulsif chimique
  ☐ Autre : ....

Coordonnées de l'éleveur

Nom et Prénom :

Adresse complète * :

Code Postal : Commune :

Tél fixe : Mobile :

Email :

France Nature Environnement 25 et toute l'équipe du programme CARELI vous remercie d'avoir répondu à ce questionnaire !

Pour toute demande d'informations :

France Nature Environnement 25
Antenne FNE du Doubs
C/O Maison de l'environnement de Franche-Comté
7 rue Voirin
25000 BESANCON
Tél : 03 81 61 36 44
mission@fne25.fr

*(à remplir si l'adresse est différente de l'adresse de l'élevage)
