# Supplementary material for: Investigating the effects of red fox management on poultry beyond the controversy, Jura Massif, France
Source: Sci Rep. 2025 Jul 19;15:26238. doi: 10.1038/s41598-025-08500-6 (PMC12276238; doi:10.1038/s41598-025-08500-6)
Supplement: Supplementary file 1 — Supplementary Material 1 [file 41598_2025_8500_MOESM1_ESM.zip › Supplementary_material_2_Fiche déclarative_degats.pdf]

Programme d'étude CARELI

Code

Fiche déclarative des dommages aux élevages avicoles

Votre élevage a subi récemment des dommages occasionnés par un prédateur. Nous vous remercions de bien vouloir remplir le questionnaire suivant.
Toutes les parties grisées de ce formulaire nous sont réservées et ne sont donc pas à renseigner.

Les informations portées sur ce formulaire font l'objet d'un traitement informatisé destiné à FNE 25 et seront traitées de façon anonymisée par l'équipe du programme CARELI. Depuis la loi "informatique et libertés" du 6 janvier 1978 modifiée, vous bénéficiez d'un droit d'accès et de rectification aux informations qui vous concernent. Si vous souhaitez exercer ce droit et obtenir communication des informations vous concernant, veuillez vous adresser à mission@fne25.fr - Représentant FNE 25 du programme CARELI.

Nom de la personne ayant renseigné cette fiche :

Date de la description : ....

Coordonnées et type de l'élevage

Nom et prénon du déclarant :

Commune :

- Type d'élevage : ☐ Professionnel ☐ Particulier

Nature des dégâts

- Indiquer dans les cases correspondantes le nombre de victimes :

	Poule	Dinde	Canard	Pintade	Caille	Oie	Pigeon	Perdrix	....
Tuée et laissée sur place									
Consommée sur place									
Emportée									
Blessée									

Circonstances de l'évènement

- Date des faits (jour / mois / année) : .... /.......................... /...........................
- Heure des faits (dans la mesure du possible) : ....
  ou choisir entre : ☐ Matin ☐ Après-midi ☐ Fin de journée ☐ journée ☐ Nuit
- Météo au moment des faits :
  ☐ Ensoleillé ☐ Nuageux ☐ Pluies ☐ Orageux ☐ Brouillard ☐ Vent ☐ Neige au sol
  ☐ Chute de neige ☐ Conditions exceptionnelles : ....

Températures et pluviométrie de Météofrance : ....

- État de la fenaison sur le territoire communale au moment des faits :
  (le point de référence étant le 1er janvier de l'année en cours)

  ☐ Pas encore commencée ☐ En cours ☐ Terminée ☐ Ne sait pas

Auteur des faits

- ☐ Belette ☐ Blaireau ☐ Martre ☐ Chat forestier ☐ Chien
  ☐ Hermine ☐ Fouine ☐ Putois ☐ Rat ☐ Renard ☐ Autre mammifère : ………………........

- ☐ Autour ☐ Busard ☐ Buse ☐ Faucon ☐ Epervier ☐ Milan ☐ Rapace indéterminé
  ☐ Pie bavarde ☐ Corneille ☐ Corbeau freux ☐ Grand Corbeau ☐ Corvidé indéterminé
  ☐ Autre oiseau : ………………….........

- ☐ Non identifié

- L'auteur des faits a été observé en flagrant délit ou en train de s'enfuir : ☐ OUI ☐ NON
- L'auteur des faits n'a pas été observé mais vous pensez qu'il s'agit bien de cet auteur : ☐ OUI ☐ NON
- Indices éventuellement trouvés sur place permettant d'identifier l'auteur des faits :
  (Ces indices doivent appartenir à l'auteur des faits et non aux victimes.)

  ☐ Excréments ☐ Traces ☐ Poils ☐ Plumes ☐ Autre : ……………..........

Caractéristiques de l'évènement

- L'auteur des faits a pénétré dans le bâtiment d'élevage (où les volailles dorment ou pondent) :
  - ☐ En creusant un trou
  - ☐ Par une ouverture laissée ouverte
  - ☐ Par un dysfonctionnement du système de fermeture
  - ☐ En détériorant une partie du bâtiment. Précisez : ....
  - ☐ Par tout autre évènement dont l'auteur des faits ne peut pas être tenu pour responsable.
  - ☐ Autre : ....
- L'auteur des faits a pénétré dans le parcours extérieur d'élevage (où les volailles peuvent sortir) :
  - ☐ En creusant un trou
  - ☐ En passant au dessus de la clôture
  - ☐ En passant à travers la clôture via un trou déjà existant
  - ☐ En détériorant la clôture
  - ☐ Par une porte, un accès, laissés ouverts
  - ☐ Par tout autre évènement dont l'auteur des faits ne peut pas être tenu pour responsable.
  - ☐ Autre : ....
- Indices ayant pu être relevés :
  - ☐ Plumes arrachées
  - ☐ Plumes coupées
  - ☐ Volaille saignée (traces de morsures au cou ou sur la tête)
  - ☐ Volaille éviscérées
  - ☐ Volaille aux yeux mangés
  - ☐ Volaille décapitée
  - ☐ Volaille sur le dos avec cage thoracique ouverte
  - ☐ Volaille consommée en commençant par le dos
  - ☐ Autre : ....

Nous vous remercions de nous communiquer, dans la mesure du possible, des photos des indices retrouvés sur place après les faits ainsi qu'une photo du ou des cadavres.

France Nature Environnement 25
et toute l'équipe du programme CARELI
vous remercie d'avoir répondu à ce questionnaire !

Pour toute demande d'informations :

France Nature Environnement 25
Antenne FNE du Doubs
C/O Maison de l'environnement de Franche-Comté
7 rue Voirin
25000 BESANCON
Tél : 03 81 61 36 44
mission@fne25.fr
