# Supplementary material for: Investigating the effects of red fox management on poultry beyond the controversy, Jura Massif, France
Source: Sci Rep. 2025 Jul 19;15:26238. doi: 10.1038/s41598-025-08500-6 (PMC12276238; doi:10.1038/s41598-025-08500-6)
Supplement: Supplementary file 1 — Supplementary Material 1 [file 41598_2025_8500_MOESM1_ESM.zip › Supplementary_material_5_models.docx]

**Supplementary material 5**

# Model for all damage categories included

## MON

summary(modM0mneg2)

## ******************************************************************
## Family: c("NBI", "Negative Binomial type I")
##
## Call: gamlss(formula = deg ~ offset(log(durobs)) + zone + re(random = ~1 |
## com), family = NBI, data = dbps[substr(dbps$zone, 1, 2) == "MO", ])
##
## Fitting method: RS()
##
## ------------------------------------------------------------------
## Mu link function: log
## Mu Coefficients:
## Estimate Std. Error t value Pr(>|t|)
## (Intercept) -6.3446 0.1657 -38.289 <2e-16 ***
## zoneMO2 -0.5769 0.2447 -2.358 0.0198 *
## ---
## Signif. codes: 0 '***' 0.001 '**' 0.01 '*' 0.05 '.' 0.1 ' ' 1
##
## ------------------------------------------------------------------
## Sigma link function: log
## Sigma Coefficients:
## Estimate Std. Error t value Pr(>|t|)
## (Intercept) -0.0126 0.3313 -0.038 0.97
##
## No. of observations in the fit: 137
## Degrees of Freedom for the fit: 2
## Residual Deg. of Freedom: 135
## at cycle: 2

Bayesian estimation (CI, credibility interval)

Estimate 95%CIlow 95%CIhigh p(coef >=0 )

(Intercept) -6.36378 -6.822093 -5.934868

zoneMO2 -0.56237 -1.187208 0.087096 0.04

## MV

summary(modMVmneg2)

## ******************************************************************
## Family: c("NBI", "Negative Binomial type I")
##
## Call:
## gamlss(formula = deg ~ offset(log(durobs)) + zone + random(as.factor(com)),
## family = NBI(), data = dbps[substr(dbps$zone, 1, 2) == "MV", ])
##
## Fitting method: RS()
##
## ------------------------------------------------------------------
## Mu link function: log
## Mu Coefficients:
## Estimate Std. Error t value Pr(>|t|)
## (Intercept) -6.7139 0.2116 -31.724 <2e-16 ***
## zoneMV2 -0.1995 0.2939 -0.679 0.499
## ---
## Signif. codes: 0 '***' 0.001 '**' 0.01 '*' 0.05 '.' 0.1 ' ' 1
##
## ------------------------------------------------------------------
## Sigma link function: log
## Sigma Coefficients:
## Estimate Std. Error t value Pr(>|t|)
## (Intercept) -0.1622 0.4121 -0.394 0.695
##

Bayesian estimation (CI, credibility interval)

Estimate 95%CIlow 95%CIhigh p(coef>=0)

(Intercept) -6.76950 -7.38926 -6.17670

zoneMV2 -0.16389 -1.03281 0.72944 0.329

# Model for damage attributed to fox only

## MON

summary(modM0mneg)

Generalized linear mixed model fit by maximum likelihood (Laplace Approximation) ['glmerMod']

Family: Negative Binomial(1.1182) ( log )

Formula: deg ~ offset(log(durobs)) + zone + (1 | com)

Data: dbpsren[substr(dbpsren$zone, 1, 2) == "MO", ]

AIC BIC logLik deviance df.resid

207.6 219.3 -99.8 199.6 133

Scaled residuals:

Min 1Q Median 3Q Max

-0.6750 -0.5204 -0.4185 -0.2033 6.0064

Random effects:

Groups Name Variance Std.Dev.

com (Intercept) 0.1978 0.4447

Number of obs: 137, groups: com, 10

Fixed effects:

Estimate Std. Error z value Pr(>|z|)

(Intercept) -7.5225 0.3259 -23.081 <2e-16 ***

zoneMO2 -0.7850 0.4735 -1.658 0.0973 .

---

Signif. codes: 0 ‘***’ 0.001 ‘**’ 0.01 ‘*’ 0.05 ‘.’ 0.1 ‘ ’ 1

Correlation of Fixed Effects:

(Intr)

zoneMO2 -0.611

Bayesian estimation (CI, credibility interval)

Estimate 95%CIlow 95%CIhigh p(coef>=0)

(Intercept) -7.6450 -8.7744 -6.6763

zoneMV2 -0.8461 -2.4095 0.6248 0.107

## MV

summary(modMVm2)

## ******************************************************************
## Family: c("PO", "Poisson")
##
## Call:
## gamlss(formula = deg ~ offset(log(durobs)) + zone + random(as.factor(com)),
## family = PO, data = dbpsren[substr(dbpsren$zone, 1, 2) == "MV", ])
##
##
## Fitting method: RS()
##
## ------------------------------------------------------------------
## Mu link function: log
## Mu Coefficients:
## Estimate Std. Error z value Pr(>|z|)
## (Intercept) -8.3147 0.3333 -24.945 <2e-16 ***
## zoneMV2 0.3378 0.4216 0.801 0.423
## ---
## Signif. codes: 0 '***' 0.001 '**' 0.01 '*' 0.05 '.' 0.1 ' ' 1
##
## No. of observations in the fit: 94
## Degrees of Freedom for the fit: 2.005446
## Residual Deg. of Freedom: 91.99455
## at cycle: 3
## ******************************************************************

Bayesian estimation (CI, credibility interval)

Estimate 95%CIlow 95%CIhigh p(coef>=0)

(Intercept) -8.43325 -9.43100 -7.6187

zoneMV2 0.42060 -0.69486 1.6676 0.773

# Model to compare protection scores

summary(glm(dbdes2_noNA$scoretot~substr(dbdes2_noNA$ID,1,3)))

Call:

glm(formula = dbdes2_noNA$scoretot ~ substr(dbdes2_noNA$ID, 1,

3))

Coefficients:

Estimate Std. Error t value Pr(>|t|)

(Intercept) 27.8657 0.7624 36.550 <2e-16 ***

substr(dbdes2_noNA$ID, 1, 3)MO2 -0.7130 1.0782 -0.661 0.509

substr(dbdes2_noNA$ID, 1, 3)MV1 -2.0250 1.2293 -1.647 0.101

substr(dbdes2_noNA$ID, 1, 3)MV2 -0.2234 1.2055 -0.185 0.853

---

Signif. codes: 0 ‘***’ 0.001 ‘**’ 0.01 ‘*’ 0.05 ‘.’ 0.1 ‘ ’ 1

(Dispersion parameter for gaussian family taken to be 41.85124)

Null deviance: 9875.9 on 236 degrees of freedom

Residual deviance: 9751.3 on 233 degrees of freedom

AIC: 1563.5

Number of Fisher Scoring iterations: 2

# Model to analyse the contribution of poultry house condition variables against fox predation

> modP<-glm(foxdeg~offset(log(durobs))+sol+ntotouv+sysferm+parcours+solparc+natureparc+hautparc+protechautparc+protecbasparc+surveil, family=poisson, data=dbes2_fox)

> modNB<-glm.nb(foxdeg~offset(log(durobs))+sol+ntotouv+sysferm+parcours+solparc+natureparc+hautparc+protechautparc+protecbasparc+surveil, data=dbes2_fox)

> AIC(modP,modNB)

df AIC

modP 11 285.6646

modNB 12 286.4983

> summary(modP)

Call:

glm(formula = foxdeg ~ offset(log(durobs)) + sol + ntotouv +

sysferm + parcours + solparc + natureparc + hautparc + protechautparc +

protecbasparc + surveil, family = poisson, data = dbes2_fox)

Coefficients:

Estimate Std. Error z value Pr(>|z|)

(Intercept) -2.531e+00 6.379e+02 -0.004 0.9968

sol 7.278e-02 8.261e-02 0.881 0.3783

ntotouv 3.491e-01 2.501e-01 1.396 0.1628

sysferm 7.790e-02 1.208e-01 0.645 0.5192

parcours 4.445e-02 1.080e-01 0.411 0.6807

solparc -6.844e+00 6.379e+02 -0.011 0.9914

natureparc 8.467e-02 2.694e-01 0.314 0.7533

hautparc 1.248e-01 1.486e-01 0.840 0.4011

protechautparc -9.785e-02 5.034e-02 -1.944 0.0519 .

protecbasparc -1.208e-01 5.524e-02 -2.187 0.0288 *

surveil 5.542e-05 1.266e-01 0.000 0.9997

---

Signif. codes: 0 ‘***’ 0.001 ‘**’ 0.01 ‘*’ 0.05 ‘.’ 0.1 ‘ ’ 1

(Dispersion parameter for poisson family taken to be 1)

Null deviance: 171.24 on 177 degrees of freedom

Residual deviance: 152.28 on 167 degrees of freedom

AIC: 285.66

Number of Fisher Scoring iterations: 13

> modP2<-glm(foxdeg~offset(log(durobs))+protechautparc+protecbasparc, family=poisson, data=dbes2_fox)

> summary(modP2)

Call:

glm(formula = foxdeg ~ offset(log(durobs)) + protechautparc +

protecbasparc, family = poisson, data = dbes2_fox)

Coefficients:

Estimate Std. Error z value Pr(>|z|)

(Intercept) -7.39802 0.13500 -54.799 <2e-16 ***

protechautparc -0.10135 0.05015 -2.021 0.0433 *

protecbasparc -0.10907 0.05379 -2.028 0.0426 *

---

Signif. codes: 0 ‘***’ 0.001 ‘**’ 0.01 ‘*’ 0.05 ‘.’ 0.1 ‘ ’ 1

(Dispersion parameter for poisson family taken to be 1)

Null deviance: 171.24 on 177 degrees of freedom

Residual deviance: 156.94 on 175 degrees of freedom

AIC: 274.32

Number of Fisher Scoring iterations: 6

Bayesian estimation (CI, credibility interval)

Estimate 95%CIlow 95%CIhigh

(Intercept) -7.4046 -7.6816 -7.14412

Beta1prothaut -0.1108 -0.2211 -0.01689

Beta2protbas -0.1183 -0.2364 -0.01749
